# Dielectric Properties and Heating Rates of Egg Components Associated with Radio Frequency and Microwave Pasteurization

**DOI:** 10.3390/foods14193287

**Published:** 2025-09-23

**Authors:** Feixue Yang, Jianhang Hu, Huijia Li, Xinyu Tang, Qisen Xiang, Xiangyu Guan, Wenhao Sun, Ping Li, Haiyan Zhang, Teng Cheng

**Affiliations:** 1 College of Food and Bioengineering, Zhengzhou University of Light Industry, Zhengzhou 450001, China; 15838256074@163.com (F.Y.);; 2 Key Laboratory of Cold Chain Food Processing and Safety Control, Ministry of Education, Zhengzhou University of Light Industry, Zhengzhou 450001, China; 3 Key Laboratory of Food Nutrition and Health in Universities of Shandong, College of Food Science and Engineering, Shandong Agricultural University, Tai’an 271018, China

**Keywords:** computer simulation, model analysis, penetration depth, *Salmonella* spp., thermal pasteurization

## Abstract

*Salmonella* spp. outbreaks associated with eggs have attracted widespread concerns about food safety. To provide necessary information for further pasteurization processes and computer simulations induced by radio frequency (RF) and microwave (MW) energy, the dielectric properties, penetration depth, and heating rates of egg white, yolk, and eggshell were measured, calculated, or fitted by regression models. The results demonstrated that both the dielectric constant and dielectric loss factor of egg white and yolk decreased dramatically with raised frequency within the RF range from 10 to 300 MHz, and then reduced slightly within the MW range from 300 to 3000 MHz. Dielectric constant, and loss factor of egg white, yolk, and eggshell increased with raised temperature. The penetration depth of egg white, yolk, and eggshell decreased with increasing of frequency. RF waves had a deeper penetration depth than that of MW waves at the same temperature. The fourth-order polynomial models provided a good fit to the experimental data with large coefficients of determination (*R*^2^ > 0.902). The heating rate of the egg samples increased with increasing RF voltage and microwave power, and the heating rate of yolk was higher than that of egg white or eggshell at the same conditions. This study offers essential data and effective guidance in developing and optimizing RF and MW pasteurization techniques for ensuring the microbial safety of eggs, using both experiments and mathematical simulations.

## 1. Introduction

Eggs are rich in high-quality protein, vitamins, phospholipids, and other nutrients, and are one of the most important sources of nutrition in the human diet [1]. The global production of eggs was approximately 85.03 million metric tons in 2022, which was mainly contributed by China (34.8%), India (7.7%), USA (7.7%), Indonesia (7.0%), Brazil (3.9%), Mexico (3.6%), and Japan (3.1%) [2]. However, it cannot be ignored that eggs can easily be contaminated by harmful microorganisms, such as *Salmonella* spp., due to the presence of these microorganisms in the hens themselves or in the environment [3]. The latest outbreak of *Salmonella* spp. in eggs infected 65 people and caused 24 people to be hospitalized, according to the CDC [4]. A significant amount of foodborne illness can be reduced by pasteurizing eggs [5]. Therefore, it is crucial to inactivate harmful microorganisms inside eggs. The composition of eggs is complex, and the pasteurization of shell eggs is more difficult [6]. Accelerated progress in computational modeling techniques enables precise parameterize of emerging thermal pasteurization platforms. This paradigm significantly improves the techno-economic viability of eliminating subsurface pathogens in egg shells without compromising functional properties of egg white or yolk microstructure.

Several methods have been investigated for pasteurization of the surface and interior of eggs [7,8,9]. Radio frequency (RF) and microwave (MW) are new electromagnetic heating pasteurization technologies, which have broad application prospects in the pasteurization of eggs [10,11,12]. When the sample is exposed to RF and MW electromagnetic waves, the electromagnetic energy causes friction between the polar molecules inside the sample, generates heat, and causes the object to heat up [13]. RF and MW heating have the characteristics of rapid heating [14,15]. As an emerging pasteurization technology, RF and MW treatments have broad prospects for the pasteurization of eggs.

Dielectric properties, penetration depth, and heating rates are critical parameters for developing computer simulation of dielectric pasteurization protocols based on the RF and MW treatment of egg shells. Food can store and dissipate electromagnetic energy (ε), which is represented by the dielectric constant (ε′) and dielectric loss factor (ε″), respectively [16,17]. The dielectric properties, dielectric constant, and dielectric loss factor can be expressed by the following formula: $\varepsilon =\varepsilon^{\prime }-j\varepsilon^{\prime \prime }\left(j=\sqrt{-1}\right)$, where $j=\sqrt{-1}$.

Many studies on agricultural products and foods have presented that the dielectric properties are functions of frequency and temperature [18,19,20]. Wei et al. (2021) investigated the dielectric heterogeneity and radio frequency differential heating of corn kernels based on multicomponent structure, and provided a good understanding of dielectric properties and the interaction between different corn components and electromagnetic fields [21]. There are also some studies on the dielectric properties of the internal components of eggs [22,23,24]. However, they are not comprehensive enough. Exploring the dielectric properties of different egg components at different frequencies and temperatures helps us to understand the interaction between shelled eggs and electromagnetic fields. The dielectric property data of each part of the egg can be used to establish a thermal simulation model of the heating of radio frequency and microwave, thereby guiding the design of industrial equipment. However, there is still a lack of research in this area. In addition, penetration depth and heating rates have also been shown to have a significant effect on pasteurization processes and computer simulations induced by RF and MW energy [25,26].

Therefore, the purposes of this study were as follows: (1) determine the dielectric properties of egg white, yolk, and eggshell within a frequency range of 10–3000 MHz at temperatures varying from 20 to 80 °C; (2) to provide empirical equations describing dielectric properties of eggs as affected by frequency and temperature; (3) to calculate the penetration depth of electromagnetic energy into eggs at the five representative frequencies (13.56, 27.12, 40, 915, and 2450 MHz); and (4) to evaluate the influence of three given RF voltages (5250, 5600, and 5950 V) and MW powers (70, 210, and 350 W) on the heating rates of egg white, yolk, and eggshell.

## 2. Materials and Methods

### 2.1. Materials and Sample Preparation

#### 2.1.1. Egg Samples

The fresh whole eggs, within three days of grading and packing, used in this study were procured from the local market and kept in a refrigerator set at 4 °C until used. They were all of large size with an average weight of 58 ± 0.32 g, among which egg white, yolk, and eggshell were 31.19 ± 1.89 g, 19.13 ± 1.73 g, and 7.92 ± 0.24 g, respectively.

#### 2.1.2. Egg White, Yolk, and Eggshell Samples

The surface of the eggs was cleaned with deionized water and then dried. After drying, the eggs were cracked carefully, and the egg white (70 g) and yolk (70 g) were collected separately in small cylindrical beakers. The egg white and yolk were stirred slowly and homogenously with a glass rod. The eggs were broken, washed with deionized water, and dried. Then, the eggshells were separated from the shell membranes and crushed into powder. The eggshell powder was placed in a small round-bottom beaker and squeezed to a density close to that of the eggshell. All measurements were made in triplicate.

### 2.2. Dielectric Properties Measurement System

**Figure 1:**
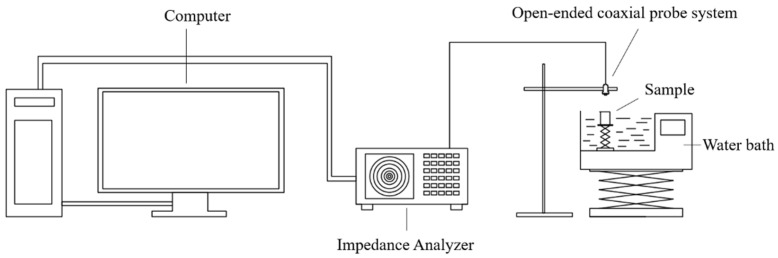
The schematic diagram of dielectric properties measurement system.

As shown in Figure 1, dielectric properties measurement system, including the impedance analyzer (Keysight Technologies, E4991B-300, Santa Rosa, USA), open-ended coaxial probe system (Keysight Technologies, 85070E-020, Santa Rosa, USA), and dielectric measurement software (Keysight Technologies, 85070E, Santa Rosa, USA), was used to measure dielectric properties of egg samples. The dielectric properties of egg white, yolk, and eggshell were measured at temperatures ranging from 20 °C (room temperature) to 60 °C (protein denaturation temperature). To avoid communication interference, the Federal Communications Commission (FCC) allocated frequencies for industrial, scientific, and medical applications, namely 13.56, 27.12, and 40.68 MHz for RF treatment, and 915 and 2450 MHz for microwave treatment. The frequency range we selected (10 MHz–3000 MHz) covers these five typical application frequencies exactly.

The instrument was warmed up for at least 30 min before the calibration and measurements were made. Calibration of the probe was performed using air and deionized water at 25 °C. The internal temperature of the samples was monitored by using a thermocouple temperature sensor (Omega, HH-25TC, California, USA). The samples were heated from 20 to 60 °C and the dielectric properties were measured while the samples were at the desired temperature of 20, 25, 30, 35, 40, 45, 50, 55, and 60 °C. The experiments were repeated three times.

### 2.3. Determination of Penetration Depth

The penetration depth (*dp*) of microwave power in a dielectric material is the depth where the incident power decreases to 1/e (e = 2.718) of its original value at the material surface. *dp* can be calculated from the following:

(1) $$d_{p}=\frac{c}{2\pi f\sqrt{2\varepsilon^{\prime }\left[\sqrt{{\left(\frac{\varepsilon^{\prime \prime }}{\varepsilon^{\prime }}\right)}^{2}+1}-1\right]}}$$

where *c* is the speed of light in the free space as 3 × 10^8^ m/s, and *f* is the frequency (Hz).

### 2.4. Regression Models for Dielectric Properties

Variance (ANOVA) was used to analyze the relationships of temperature (*T*, °C) with the dielectric property of egg white, yolk, and eggshell at five frequencies (13.56, 27.12, 40, 915, and 2450 MHz), and the significant terms on dielectric property using Design Expert 13.0. (Stat-Ease, Inc. Minneapolis, MN, USA). The fitted polynomial models used in regression analysis were as follows:

(2) $$\varepsilon^{\prime }=a_{0}+a_{1}T+a_{2}T^{2}+a_{3}T^{3}+a_{4}T^{4}$$

(3) $$\varepsilon^{\prime \prime }=a_{0}+a_{1}T+a_{2}T^{2}+a_{3}T^{3}$$

(4) $$\begin{array}{l} \varepsilon^{\prime }or\varepsilon^{\prime \prime }=a_{0}+a_{1}T+a_{2}F+a_{12}TF+a_{11}T^{2}+a_{22}F^{2}+a_{112}T^{2}F+a_{122}TF^{2}+a_{111}T^{3}\\ +a_{222}F^{3}+a_{1122}T^{2}F^{2}+a_{1112}T^{3}F+a_{1222}TF^{3}+a_{1111}T^{4}+a_{2222}F^{4} \end{array}$$

(5) $$\begin{array}{l} \varepsilon^{\prime }or\varepsilon^{\prime \prime }=a_{0}+a_{1}T+a_{2}F+a_{12}TF+a_{11}T^{2}+a_{22}F^{2}+a_{112}T^{2}F\\ +a_{122}TF^{2}+a_{111}T^{3}+a_{222}F^{3} \end{array}$$

where a_0_, a_1_, a_2_, a_3_, a_4_, a_12_, a_11_, a_22_, a_112_, a_122_, a_111_, a_222_, a_1122_, a_1112_, a_1222_, a_1111_, and a_2222_ are regression coefficients.

### 2.5. RF and MW Treatments

#### 2.5.1. RF Treatments

**Figure 2:**
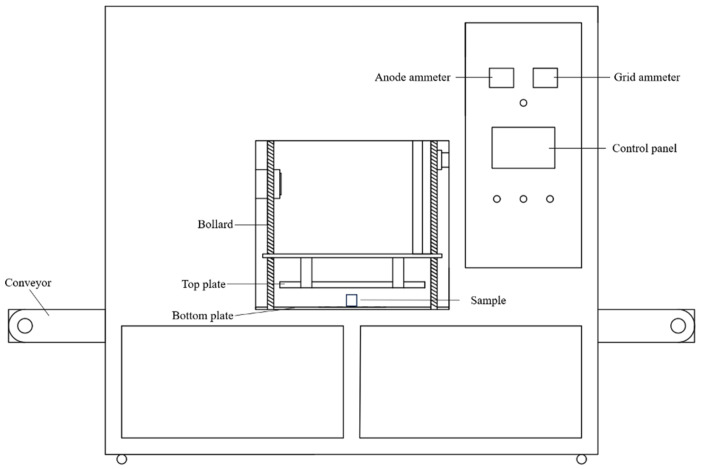
Schematic of the 6 kW, 27.12 MHz RF processing equipment (HGJL-6RFS).

A 6 kW, 27.12 MHz RF device (HGJL-6RFS, HGJL, Anhui, China) was used for thermal treatment (Figure 2). Egg white, yolk, and eggshell samples were placed between the upper and lower plates, respectively. The electrode gap is adjusted to 8 cm. The RF voltage was adjusted to 75%, 80%, and 85% of the maximum anode voltage (7000 V), namely, 5250, 5600, and 5950 V, respectively. The temperature-measuring optical fiber (SR-C2A6F20, Skyray, Fujian, China) was inserted into the geometric center of the sample, and the temperature changes inside the sample were monitored in real time [11].

#### 2.5.2. MW Treatments

A 700 W, 2450 MHz MW device (M22J, Midea, Guangdong, China) was used for thermal treatment. Egg white, yolk, and eggshell samples were placed in the processing chamber, and the temperature was measured using thermocouples at specific intervals.

### 2.6. Determination of Heating Rates of Egg White, Yolk, and Eggshell

In this experiment, egg white, yolk, and eggshell were heated from 20 °C to 60 °C in the RF and the microwave oven, respectively. The effects of three given RF voltages (5250, 5600, and 5950 V) and microwave powers (70, 210, and 350 W) were evaluated on heating rates.

### 2.7. Data Analysis

The results were expressed as means ± standard deviations over three replicates. Differences were determined by the analysis of variance followed by Duncan’s multiple range test and considered significant at *p* < 0.05. Data were analyzed using the statistical software SPSS 26.0 version (IBM, Armonk, NY, USA) and Microsoft Excel variance procedure (Microsoft Office Excel, 2021).

## 3. Results

### 3.1. Effects of Frequency and Temperature on Dielectric Properties

#### 3.1.1. Effects of Frequency and Temperature on Dielectric Constant

**Figure 3:**
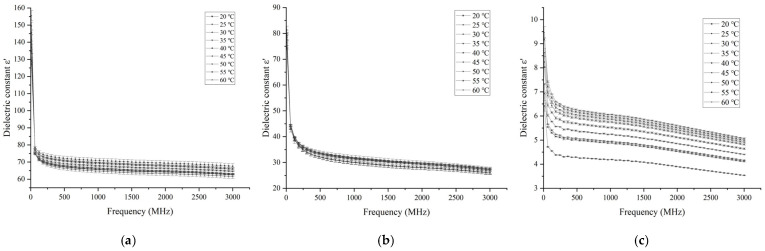
Effects of frequency and temperature variation on the dielectric constants of egg white (**a**), yolk (**b**), and eggshell (**c**).

The effect of frequency and temperature variation on the dielectric constant of different parts of the egg is shown in Figure 3. The dielectric constants of egg white, yolk, and eggshell all show a downward trend with the increase in frequency, and the decrease in the dielectric constants of egg white and yolk becomes smaller with the increase in frequency. The rate decreases significantly at a frequency around 70 MHz. Similar results have been reported in duck egg white [24], fish [27], wheat flour [28], and chili powder [13], etc.

The dielectric constant of egg white shows a trend of decreasing first and then increasing with increasing temperature at frequencies of 13.56, 27.12, and 40 MHz, while it shows a trend of decreasing with increasing temperature at frequencies of 915 and 2450 MHz. Wang et al. [23] and Soon et al. [22] observed similar behaviors in egg whites. They reported that the trend in egg white ε′ with temperature in the radiofrequency heating frequencies reversed at somewhere about 50 °C. They speculated that the coagulation of egg whites is likely responsible for the trend reversal.

The trends of the dielectric constant of yolk changing with temperature are opposite at radio frequency and microwave frequency. The dielectric constant of yolk shows a slow upward trend as the temperature increases at 13.56, 27.12, and 40 MHz frequencies. It changes more steadily with the temperature at 915 and 2450 MHz frequencies, but decreases significantly when the temperature rises to 45 °C. The dielectric constant of the yolk was less sensitive to temperature than the egg white. Soon et al. [22] reported a similar behavior for yolk.

The dielectric constant of eggshell increases with increasing temperature at frequencies of 13.56, 27.12, 40, 915, and 2450 MHz, and the rate increases at frequencies of 13.56, 27.12, and 40 MHz were higher than that at frequencies of 915 and 2450 MHz. A similar trend was observed in the study of Soon et al. [22]. However, there are also different studies that believe that the dielectric loss factor of eggshell is not related to temperature [29]. Different eggshell processing methods may be responsible for this result.

At each measured frequency and temperature, the dielectric constant of egg white was higher than that of yolk and eggshell, among which the dielectric constant of eggshell was the lowest.

#### 3.1.2. Effects of Frequency and Temperature on Loss Factor

**Figure 4:**
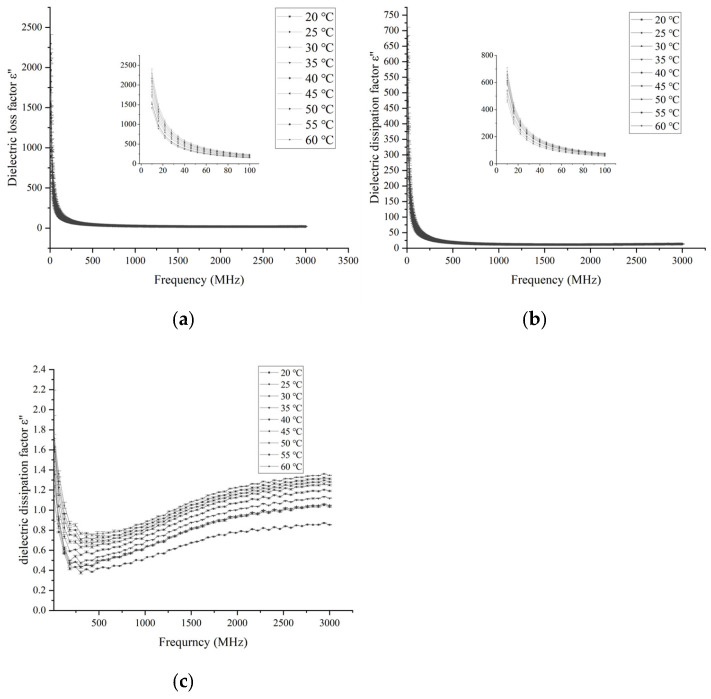
Effects of frequency and temperature variation on dielectric loss factor of egg white (**a**), yolk (**b**), and eggshell (**c**).

The effect of frequency variation on the dielectric loss factor of different parts of the egg is shown in Figure 4. The dielectric loss factor of egg white and yolk both show a decreasing trend with increasing frequency. Similarly to the change law of dielectric constant, it decreases sharply before 70 MHz and then decreases slowly. In contrast, the dielectric loss factor of eggshell decreases first and then increases with frequency. The change pattern of ε″ of eggshell with frequency is different from that of egg white and yolk. A similar phenomenon was also observed in egg white powder at 20 °C in the study of Boreddy et al. [30]. Similarly, Zhu et al. [31] also observed a similar result at 20 °C for compressed chestnut flour at a lower frequency.

The temperature variations in the dielectric loss factor of egg white and yolk at 13.56, 27.12, 40, 915, and 2450 MHz are similar. The dielectric loss factor increases with the increase in temperature at 13.56, 27.12, and 40 MHz, but has no obvious change at 915 and 2450 MHz. The dielectric loss factor of eggshell at frequencies of 13.56, 27.12, 40, 915, and 2450 MHz shows an increasing trend with increasing temperature. Moreover, the dielectric loss factor of egg white at various frequencies and temperatures is significantly higher than that of yolk and eggshell. Similar results were observed for egg white in the study by Boreddy et al. [30], where the variation in dielectric loss factor with temperature was greater at RF frequencies than at microwave frequencies. The reason for this phenomenon may be that the dipole loss components contributed by free water are different before and after 100 MHz [23].

### 3.2. Penetration Depth

**Figure 5:**
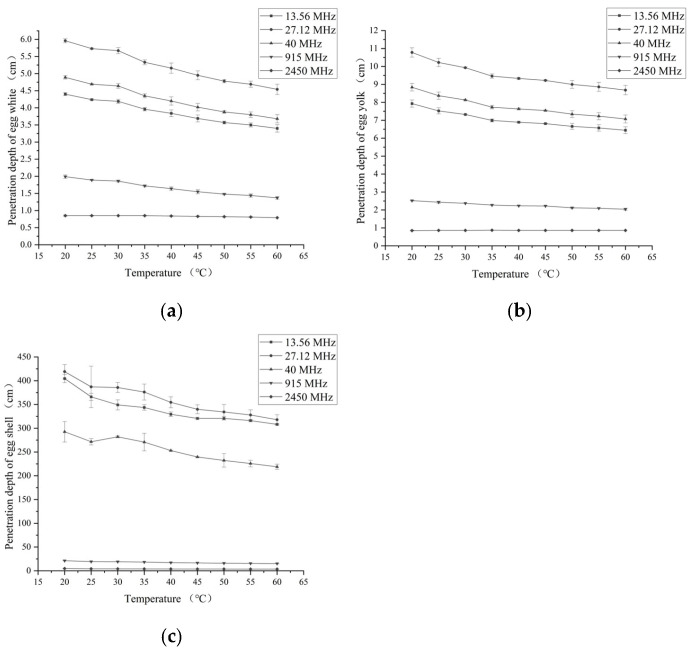
The variation in the penetration depth of egg white (**a**), yolk (**b**) and eggshell (**c**) as a function of temperature at five specific frequencies.

The penetration depths of egg whites, yolk, and eggshell at five selected frequencies and five temperatures are shown in Figure 5. The penetration depths of egg white, yolk, and eggshell with a temperature of 20 °C were 5.96 and 0.85 cm, 10.78 and 0.85 cm, 419.24, and 4.65 cm for 27.12 and 2450 MHz, respectively. The penetration depths of egg white, yolk, and eggshell at each specific frequency all tended to decrease with increasing temperature, except for the penetration depth of yolk at a frequency of 2450 MHz. The penetration depths of egg whites, yolk, and eggshell with a temperature of 60 °C were 4.54 and 0.79 cm, 8.68 and 0.86 cm, 317.98 and 3.52 cm, respectively, for two frequencies of 27.12 and 2450 MHz. The egg white, yolk, and eggshell showed a much higher penetration depth at RF than MW at the same temperature. Similar results were found in different studies of other materials [32,33,34].

### 3.3. Regression Models for Dielectric Properties of Egg White, Yolk, and Eggshell

As shown in Table 1, regression coefficients of the polynomial model (Equation (2)) to predict the dielectric properties of egg white, yolk, and eggshell as a function of temperature (20–60 °C) at five given frequencies (13.56, 27.12, 40, 915, and 2450 MHz). The relationship between temperature and dielectric constant can be described by fourth-order polynomial models. The relationship between the temperature and loss factor can also be described by fourth-order polynomial models. The regression equations all reached a high coefficient of determination with a *R^2^* greater than 0.902.

**Figure 6:**
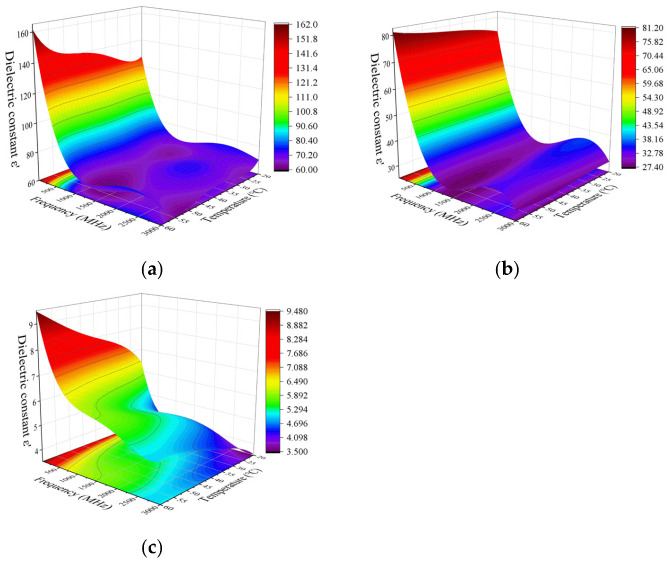
The obtained dielectric constants of egg white (**a**), yolk (**b**) and eggshell (**c**) samples as function of temperature over frequency and a temperature range from 20 to 60 °C.

**Figure 7:**
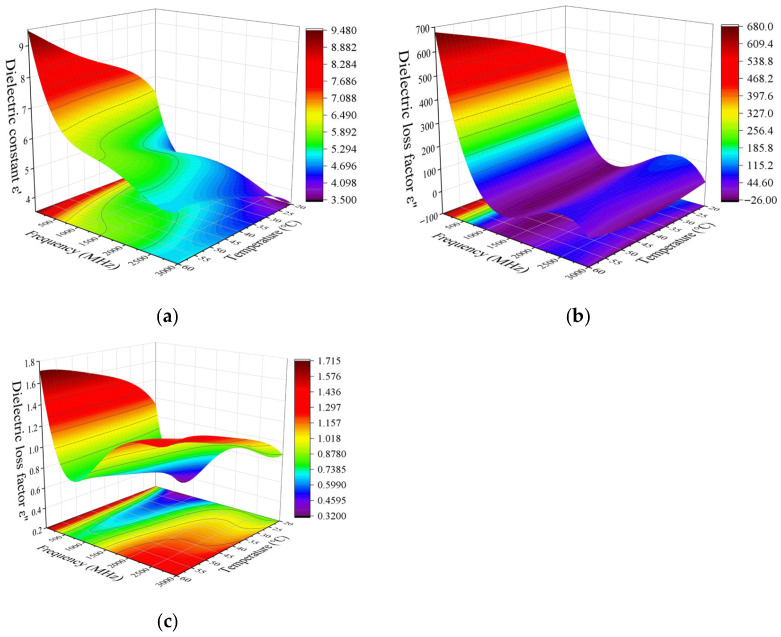
The obtained dielectric loss factor of egg white (**a**), yolk (**b**) and eggshell (**c**) samples as a function of temperature over frequency and a temperature range from 20 to 60 °C.

Figure 6 and Figure 7 show the dielectric property of egg white (a), yolk (b), and eggshell (c) as a function of frequencies and temperatures. The data in Figure 6 and Figure 7 were analyzed using Design-Expert 13 to obtain regression models describing the dielectric constants of egg white, yolk, and eggshell samples as a function of frequency and temperature. As shown in Figure 6a–c, when the temperature was kept constant, the dielectric constant of egg white, yolk, and eggshell gradually decreases with increasing frequency. The gradient of the contour line in the frequency direction changed significantly, which indicated that frequency has a greater effect on the dielectric constant of egg white, yolk, and eggshell than temperature. Differently, the gradient of the contour line of the dielectric constant of eggshell (Figure 6c) in the frequency direction changed at a slower pace with increasing temperature.

As shown in Figure 7a,b, when the temperature was kept constant, the dielectric loss factor of egg white and yolk gradually decreased with increasing frequency. The gradient of the contour line in the frequency direction changed significantly, which indicated that frequency has a greater effect on the dielectric loss factor of egg white and yolk than temperature. Differently, the gradient of the contour line of the dielectric loss factor of egg white (Figure 7a) in the frequency direction changed at a slower pace with increasing temperature. It can be seen from Figure 7c that the influence of frequency and temperature interaction on the dielectric loss factor of eggshell was distributed in a saddle point. The 3D surface plot shows an initial downward slope, followed by an upward movement, and then a final downward trajectory with increasing frequency.

Table 2 shows the regression coefficients of the polynomial model (Equation (3)) to predict the dielectric properties of egg white, yolk and eggshell as a function of given frequencies and temperatures. Table 3 shows the significance of probability of regressed models of Eqs. As shown in Table 2 and Table 3, the fourth-order polynomial models provided a good fit to the experimental data at the significance level of 0.0001 and with a value for the coefficient of determination of greater than 0.989. It suggests that these models can be used to precisely estimate the dielectric property value of egg white, yolk, and eggshell from known temperatures and frequencies.

### 3.4. Heating Rates of Egg White, Yolk, and Eggshell Induced by RF and MW Treatments

**Figure 8:**
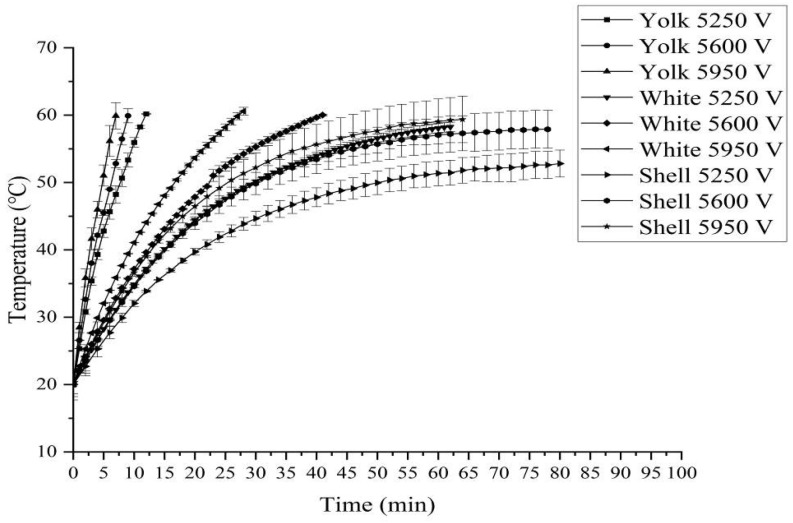
Temperature–time history of egg white, yolk and eggshell induced by RF treatment.

**Figure 9:**
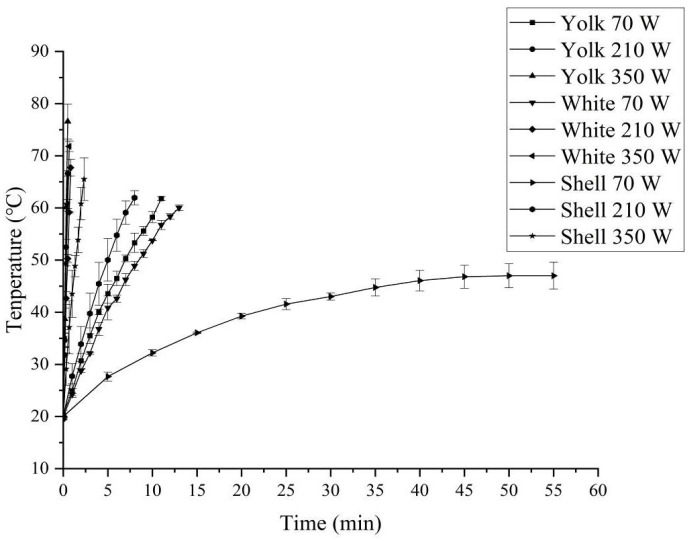
Temperature–time history of egg white, yolk and eggshell induced by MW treatment.

Figure 8 and Figure 9 show the temperature–time history of egg white, yolk, and eggshell samples induced by RF and MW treatments. It was shown from Figure 8 that the heating rates of egg white, yolk, and eggshell samples during RF treatment increased with increasing RF voltage and microwave power, and the heating rate of yolk was higher than that of egg white or eggshell. The possible reasons might be the higher dielectric loss factors of yolk being larger than that of egg white at the same temperature and frequency. A similar result was observed in the study of Zhu et al. [35]. The reason for this may be that the penetration depth of yolk is much higher than that of egg white, and the dielectric constant and dielectric loss factor of yolk and egg white are much higher than those of eggshell. Furthermore, for all tested RF anode high voltages and microwave powers, the heating rate is proportional to the RF anode high voltage and microwave power.

## 4. Conclusions

Dielectric properties of egg white, yolk, and eggshell were affected by temperature and frequency. Dielectric constant, loss factor, egg white, yolk, and eggshell increased with raised temperature. The penetration depth of egg white, yolk, and eggshell decreased with increasing frequency and made the RF heating more effective for pasteurizing bulk and thick materials as compared with the MW method. The established regression models with experimental results could predict both dielectric constants and loss factors with large coefficients of determination (*R*^2^ > 0.902). Heating rates of egg white, yolk, and eggshell samples increased with increasing RF voltage and microwave power, and the heating rate of yolk was higher than that of egg white or eggshell at the same conditions. Consequently, this study offers new information on the dielectric properties of egg, which can be useful to establish a heat simulation model of egg heating by RF and MW energy at laboratory, pilot, and industrial scales.

## Figures and Tables

**Table 1:** Regression coefficients of the polynomial model (Equation (2)) to predict the dielectric properties of egg white, yolk, and eggshell as a function of temperature (20–60 °C) at five given frequencies.

		13.56 MHz		27.12 MHz		40 MHz		915 MHz		2450 MHz	
		ε′	ε″	ε′	ε″	ε′	ε″	ε′	ε″	ε′	ε″
Egg white	a_0_	116.87	994.66	94.285	573.02	86.153	399.85	56.678	23.505	53.906	20.43
	a_1_	−2.0969	8.909	−0.7221	−10.762	−0.3462	−7.3228	2.0436	−0.2574	2.0877	−0.837
	a_2_	0.0832	0.8329	0.0259	0.4754	0.0086	0.3268	−0.971	0.0119	−0.0992	0.001
	a_3_	−0.0014	−0.0066	−0.0005	−0.0037	−0.0002	−0.0026	0.0017	−9 × 10^−5^	0.0018	3 × 10^−6^
	a_4_	9 × 10^−6^		4 × 10^−6^		2 × 10^−6^		1 × 10^−5^		−1 × 10^−5^	
	*R* ^2^	0.9934	0.9956	0.9874	0.9955	0.9837	0.9955	0.9854	0.9958	0.9829	0.9665
Yolk	a_0_	81.739	95.578	73.246	59.928	68.552	44.514	41.102	8.2363	35.419	11.414
	a_1_	−2.9118	14.791	−2.7951	8.4916	−2.7553	5.9678	−1.2593	0.2579	−1.018	0.0981
	a_2_	0.1331	−0.2664	0.1255	−0.1531	0.1229	−0.1078	0.0595	−0.0045	0.0507	−0.0028
	a_3_	−0.0025	0.0019	−0.0023	0.0011	−0.0022	0.0008	−0.0011	3 × 10^−5^	−0.001	2 × 10^−5^
	a_4_	2 × 10^−5^		1 × 10^−5^		1 × 10^−5^		8 × 10^−6^		7 × 10^−6^	
	*R* ^2^	0.9887	0.9950	0.9393	0.9951	0.9043	0.9951	0.9233	0.9974	0.9644	0.9020
Eggshell	a_0_	9.8177	0.5054	6.9896	0.9156	6.6067	1.8662	−3.8040	0.2618	−2.7466	0.1931
	a_1_	−0.3857	0.0416	−0.27764	−0.009	−0.2962	−0.09012	0.8216	0.0133	0.6532	0.0415
	a_2_	−0.01355	−0.0006	0.01496	8 × 10^−4^	0.1604	0.00276	−0.0298	−6 × 10^−5^	−0.0232	−5.9 × 10^−4^
	a_3_	−1.4 × 10^−4^	4.7 × 10^−6^	−0.00025	−7.6 × 10^−6^	−2.7 × 10^−4^	−2.3 × 10^−5^	5 × 10^−4^	5.8 × 10^−8^	3.9 × 10^−4^	3.4 × 10^−6^
	a_4_	2.6 × 10^−7^		1.4 × 10^−6^		1.6 × 10^−6^		−3 × 10^−6^		−2.4 × 10^−6^	
	*R* ^2^	0.9912	0.9820	0.9836	0.9970	0.9972	0.9886	0.9933	0.9972	0.9927	0.9936

**Table 2:** Regression coefficients of the polynomial model (Equation (3)) to predict the dielectric properties of egg white, yolk and eggshell as a function of frequency (10–3000 MHz) and temperatures (20–60 °C).

	Egg White		Yolk		Eggshell	
	ε′	ε″	ε′	ε″	ε′	ε″
a_0_	347.7161	7919.58866	62.85377	283.4594	−2.24302	−1.09470
a_1_	−26.73673	−800.33164	0.940589	12.49945	0.835286	0.01216
a_2_	−0.112731	−2.79984	−7.9989 × 10^−2^	−0.87386	−6.97 × 10^−3^	2.502 × 10^−3^
a_12_	−1.324 × 10^−3^	−4.2245 × 10^−2^	−3.13 × 10^−4^	−8.74 × 10^−3^	−3.5 × 10^−5^	−3.3 × 10^−3^
a_11_	1.14605	34.6736	−2.0246 × 10^−2^	−0.146662	−2.7204 × 10^−2^	−6.749 × 10^−3^
a_22_	9.7 × 10^−5^	2.563 × 10^−3^	5 × 10^−5^	6.28 × 10^−4^	7.55381 × 10^−6^	3.30809 × 10^−6^
a_112_	5.31364 × 10^−6^	3.1 × 10^−4^	8.58045 × 10^−7^	4 × 10^−5^	1.30068 × 10^−6^	4.55551 × 10^−7^
a_122_	6.25052 × 10^−7^	1.6 × 10^−5^	6.01474 × 10^−8^	1.26969 × 10^−6^	−1.90438 × 10^−8^	7.62115 × 10^−9^
a_111_	−2.0507 × 10^−2^	−0.620937	1.65 × 10^−4^	8.62 × 10^−4^	4.07 × 10^−4^	1.01 × 10^−4^
a_222_	−3.25677 × 10^−8^	−8.70530 × 10^−7^	−9.21494 × 10^−9^	−1.20518 × 10^−7^	−2.93614 × 10^−9^	−1.37577 × 10^−9^
a_1122_	1.64497 × 10^−9^	1.9443 × 10^−8^			1.9815 × 10^−10^	−5.58362 × 10^−11^
a_1112_	−1.04689 × 10^−7^	−3.30906 × 10^−6^			−1.72482 × 10^−8^	−2.09252 × 10^−9^
a_1222_	−1.24201 × 10^−10^	−2.76264 × 10^−9^			1.94927 × 10^−12^	−3.1911 × 10^−13^
a_1111_	1.33 × 10^−4^	3.993 × 10^−3^			−2.18433 × 10^−6^	−5.71768 × 10^−7^
a_2222_	3.83130 × 10^−12^	1.01493 × 10^−10^			3.93449 × 10^−13^	1.86083 × 10^−13^
*R* ^2^	1	1	0.9912	0.9897	0.9998	0.9997

**Table 3:** Significance of probability (p) of regressed models of Eqs.

Variances and *R*^2^	Egg White	Yolk	Eggshell
T	<0.0001	0.4953	<0.0001
F	<0.0001	0.2463	0.1138
TF	0.0073	0.0737	0.0056
T^2^	<0.0001	0.8262	0.8927
F^2^	<0.0001	<0.0001	0.0002
T^2^F	<0.0001	0.7940	0.0020
TF^2^	<0.0001	0.2411	<0.0001
T^3^	<0.0001	0.7396	0.0007
F^3^	<0.0001	- ^a^	<0.0001
T^2^F^2^	<0.0001	-	0.0009
T^3^F	0.0640	-	0.0263
TF^3^	<0.0001	-	0.2480
T^4^	<0.0001	-	0.0392
F^4^	<0.0001	-	<0.0001
Model	<0.0001	<0.0001	<0.0001
*R* ^2^	1	0.9912	0.9998

^a^ Dashes in the table indicate the term was not included in the regressed model.

## Data Availability

The original contributions presented in the study are included in the article, further inquiries can be directed to the corresponding author.
